# Frequent and Distinct Aberrations of DNA Methylation Patterns in Fibrolamellar Carcinoma of the Liver

**DOI:** 10.1371/journal.pone.0013688

**Published:** 2010-10-29

**Authors:** Wolfgang Tränkenschuh, Florian Puls, Matthias Christgen, Cord Albat, Albert Heim, Jeanette Poczkaj, Peer Fleming, Hans Kreipe, Ulrich Lehmann

**Affiliations:** 1 Institute of Pathology, Medizinische Hochschule Hannover, Hannover, Germany; 2 Institute of Virology, Medizinische Hochschule Hannover, Hannover, Germany; The University of Hong Kong, China

## Abstract

**Background:**

Gene silencing due to aberrant DNA methylation is a frequent event in hepatocellular carcinoma (HCC) and also in hepatocellular adenoma (HCA). However, very little is known about epigenetic defects in fibrolamellar carcinoma (FLC), a rare variant of hepatocellular carcinoma that displays distinct clinical and morphological features.

**Methodology/Principal Findings:**

We analyzed the methylation status of the *APC, CDH1, cyclinD2, GSTπ1, hsa-mir-9-1, hsa-mir-9-2*, and *RASSF1A* gene in a series of 15 FLC and paired normal liver tissue specimens by quantitative high-resolution pyrosequencing. Results were compared with common HCC arising in non-cirrhotic liver (n = 10). Frequent aberrant hypermethylation was found for the *cyclinD2* (19%) and the *RASSF1A* (38%) gene as well as for the microRNA genes *mir-9-1* (13%) and mir-9-2 (33%). In contrast to common HCC the *APC* and *CDH1* (E-cadherin) genes were found devoid of any DNA methylation in FLC, whereas the *GSTπ1* gene showed comparable DNA methylation in tumor and surrounding tissue at a moderate level. Changes in global DNA methylation level were measured by analyzing methylation status of the highly repetitive LINE-1 sequences. No evidence of global hypomethylation could be found in FLCs, whereas HCCs without cirrhosis showed a significant reduction in global methylation level as described previously.

**Conclusions:**

FLCs display frequent and distinct gene-specific hypermethylation in the absence of significant global hypomethylation indicating that these two epigenetic aberrations are induced by different pathways and that full-blown malignancy can develop in the absence of global loss of DNA methylation. Only quantitative DNA methylation detection methodology was able to identify these differences.

## Introduction

Inactivation of tumor suppressor genes by aberrant methylation of cytosine residues in the promoter region is an important molecular alteration contributing to the development and progression of malignant tumors [1]. It can already be found in pre-malignant lesions and in-situ carcinomas indicating that this epigenetic alteration is an early event in carcinogenesis [2]. In colonic carcinoma acquired genetic and epigenetic defects complement one another in the process of malignant transformation [3]. The diagnostic and prognostic potential of altered DNA methylation patterns is currently being unraveled [4].

**Figure 1:**
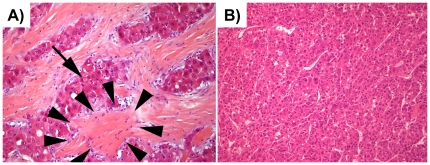
Representative histology of FLC and common HCC without cirrhosis. Representative histology of fibrolamellar carcinoma (A) and common hepatocellular carcinoma in non-cirrhotic liver (B). FLCs show large eosinpphilic tumor cells containing cytoplasmic globuli (arrow). There are abundant collagenous bands (arrowheads) separating nests of tumor cells. HCC of common type show solid nests and trabecules of smaller and paler cells without formation of collagenous bands (HE stained, original magnification A): 200×, B): 100×).

Aberrant DNA methylation is a well described phenomenon in common hepatocellular carcinoma [5] and also in hepatocellular adenoma [6]. However, very little is known about epigenetic defects in fibrolamellar carcinoma (FLC), a rare variant of hepatocellular carcinoma that displays unique clinical and morphologicall features [7], [8]. FLC occurs in the absence of chronic liver disease in children and young adults and is characterized by large eosinophilic tumor cells and abundant deposition of collagen between tumor cells (**Figure 1**). The few existing studies of genetic defects in FLCs indicate that chromosomal instability is a rare event in FLCs and that mutations frequently found in common HCC (e.g., in the *TP53* or the *CTNNB1* gene) occur at a much lower frequency if at all [9], [10]. We performed epigenetic profiling of a series of FLCs (n = 15) in comparison to common HCC arising in non-cirrhotic livers. For this purpose the global methylation level as well as gene-specific hypermethylation at 7 loci was assessed using quantitative pyrosequencing methodology.

## Results

### Selection of patients and genes under study

Upon review 15 cases of FLC were identified from the archives of the Institute of Pathology, Medizinische Hochschule Hannover. 10 cases of common HCC arising in non-cirrhotic livers were used as a control group (**Figure 1**). Patient age, sex and tumor stage are summarized in **Table 1**.

**Table 1:** Overview of patients.

FLC cases							
no.	sex	age	UICC-classification	Vascular invasion	AFIP Grade	HBV	HCV
1	male	26	pT4, pN1			neg.	n/a
2	male	20	pT3, pN1	present		neg.	neg.
3	male	20	pT4, pN1, pM1	present		neg.	neg.
4	male	39	pT1, pN0	absent		n/a	n/a
5	male	19	pT2, pNx	present		neg.	neg.
6	male	24	pT3, pN1, pM1	present		neg.	neg.
7	female	15	pT4, pN1			neg.	neg.
8	female	32	pT1, pNx	absent		neg.	neg.
9	male	19	pT3, pN1	present		neg.	neg.
10	female	28	pT1, pNx	absent		n/a	n/a
11	female	13	pT3, pN0, pM1	present		neg.	neg.
12	male	28	pT3, pN1, pM1	present		neg.	neg.
13	female	36	pT1, pN0	absent		HBV+	neg.
14	female	13	pT1, pNx	absent		neg.	neg.
15	male	22	pT1, pN0	absent		neg.	neg.

Common HCC without cirrhosis							
1	male	82	pT3, pNx	present	G3	n/a	n/a
2	female	80	pT2, pNx	present	G2	neg.	neg.
3	female	66	pT2, pN0	present	G2	n/a	n/a
4	female	69	pT1, pN0	absent	G3	neg.	neg.
5	male	67	pT1, pNx	absent	G2	neg.	neg.
6	male	84	pT3, pNx	present	G3	neg.	neg.
7	male	58	recurrence			neg.	neg.
8	male	61	pT2, pNx	present	G2	neg.	neg.
9	male	61	pT2, pNx	present	G2	neg.	neg.
10	male	78	pT1, pNx	absent	G2	neg.	HCV+

The methylation status of the following loci was analyzed in a series of 15 FLC samples and the surrounding normal appearing tissue employing quantitative high-resolution pyrosequencing technology: *APC, CDH1, cyclinD2, ESR1, GSTπ1, LINE-1, MINT31, hsa-mir-9-1, hsa-mir-9-2, RASSF1A, SFRP1, SOCS-1*. With the exception of the microRNA genes these loci are reported to be frequently hypermethylated in common hepatocellular carcinoma in more than one study (see [5] and references therein). Common HCC was chosen as a reference because HCC and FLC are regarded as to arise not only in the same organ but also in the very same cell type. Aberrant hypermethylation of microRNA genes *hsa-mir-9-1* and *hsa-mir-9-2* in liver tumors has been recently discovered in our group (Albat and Lehmann, in preparation). Gen-specific hypermethylation very often takes place in the context of a generalized hypomethylation ([11] and the methylation status of the highly repetitive *LINE-1* sequence is a suitable surrogate marker for assessing this global loss of methylation [12]. Therefore, methylation analysis of *LINE-1* was included.

Since fibrolamellar carcinoma is a rare subtype of liver carcinoma [7], several specimens were quite old (up to 20 years) and collected under non-standardized conditions. Therefore, yield and quality of genomic DNA extracted from the paraffin blocks was highly variable within this series and for several loci only a subset of samples gave reproducible results. For these reasons, the further analysis and discussion focus on the following loci: *APC, CDH1, cyclinD2, GSTπ1, LINE-1, hsa-mir-9-1, hsa-mir-9-2*, and *RASSF1A*.

**Figure 2:**
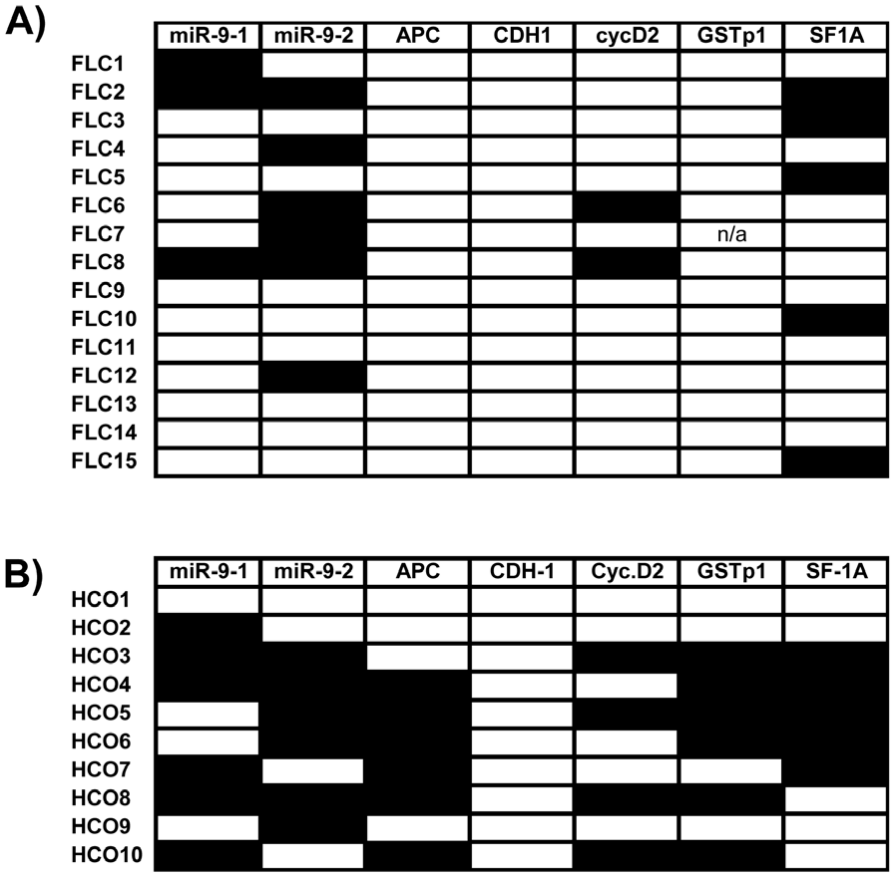
Summary of methylation data for FLC and HCC without cirrhosis. Frequent aberrant hypermethylation in FLC is obvious. A black box indicates “hypermethylated” according to the stringent threshold definition (mean of the control group plus 2× STD, see text for details).

For these 8 loci high-quality pyrosequencing data could be obtained for all specimens with only very few exceptions: The results of 399 out of 400 measurements (99.8%) are summarized in **Figures 2** **and** **3**.

**Figure 3:**
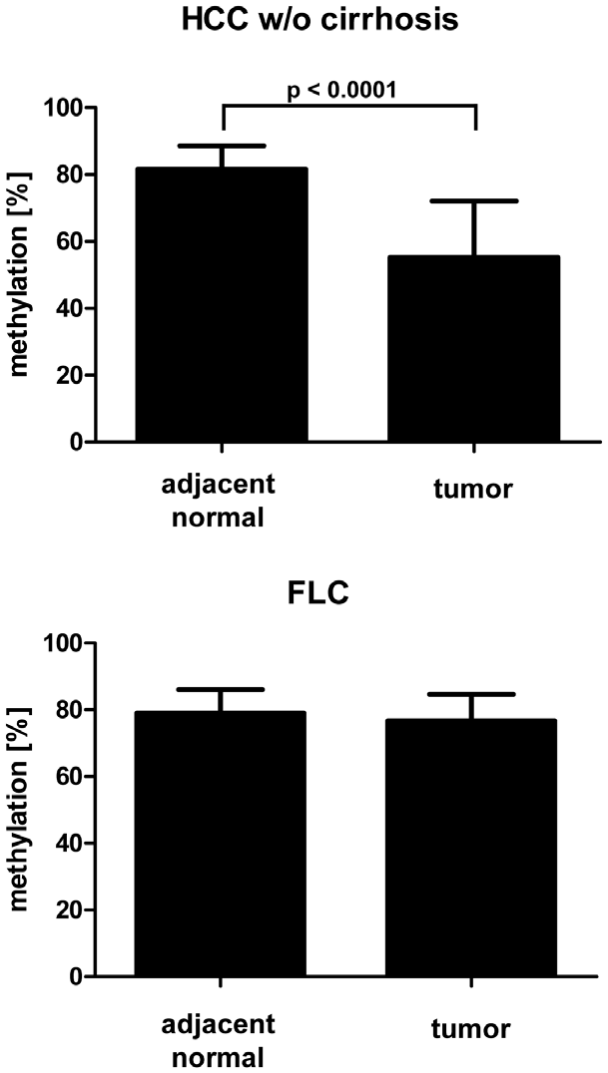
Global methylation level in FLC and HCC without cirrhosis. The Methylation level of LINE-1 sequences was measured quantitatively using pyrosequencing. The Methylation level of these repetitive elements reflects very well the overall methylation level of the genome [12].

### Definition of “hypermethylation”

DNA methylation levels for all genes under study displayed a quite high variation in the non-tumorous adjacent tissue. The range of variation ranged from 6 percentage points for the *CDH1 (E-cadherin)* gene (2–8%) to up to 49% percentage points for *GSTπ1* gene (7–56%). Therefore, two different definitions for scoring a tumor sample as “hypermethylated” were applied:

a) “hypermethylated” is defined by levels in excess of two standard deviations above the mean of the control group of adjacent non-tumorous liver tissue (“mean of control group +2× standard deviation”, [6]).

b) “hypermethylated” is defined by levels of methylation 50% higher than in the corresponding non-neoplastic liver tissue from the same patient. In order to avoid over-interpretation of data due to background fluctuations (e.g., comparing 6% in the tumor fraction with 3.5% in the adjacent normal tissue fraction) only those samples were scored “hypermethylated” in which the methylation level in the tumor fraction were above 10%. Definition a) is much more stringent, because the above mentioned high variation in the control group of normal appearing adjacent non-tumorous specimens causes a high standard deviation and thereby a high threshold value. For this reason, this threshold setting may conceal significant differences between normal and tumor for individual cases. For example, methylation level of the *cyclinD2* gene in the tumor fraction of FLC 15 is 18.8%, in the surrounding normal tissue only 5.7%. Both values are below the threshold defined following definition a) (27.8% for *cyclinD2* in FLC) but are clearly indicating increased DNA methylation in the tumor tissue (with statistical significance).

For a detailed comparison of the results using both definitions see **Figure S1**.

### Hypermethylation of *cyclinD2* and *RASSF1A* in FLC

Aberrant hypermethylation of the *cyclinD2* and the *RASSF1A* gene has been described for common hepatocellular carcinoma arising in cirrhotic liver tissue [13] as well as in many other carcinomas, including breast, gastric, and colon carcinoma [14].

In fibrolamellar carcinoma these two genes are also frequent targets of aberrant hypermethylation. Both genes were found to be hypermethylated in 2/15 (13.3%) and 5/15 (33.3%) of FLC cases, respectively. There were no statistical significant differences in comparison to the control group of common HCC (*cyclinD2*: 4/10, *RASSF1A*: 6/10, p = 0.18 and 0.24, respectively, Fisher's exact test, two-sided).

### Hypermethylation of microRNA genes *hsa-mir-9-1* and *hsa-mir-9-2* in FLC

Aberrant hypermethylation of microRNA genes has been reported for several human malignancies, including colon cancer [15], breast cancer [16], acute lymphoblastic leukemia [17], and chronic myelogenous leukemia [18]. So far, only two studies reported microRNA gene hypermethylation in liver tumors: according to Datta et al., 2 out of 4 HCC specimens tested showed hypermethylation of the *hsa-mir-1* gene [19]. In a more comprehensive study, Furuta et al. could demonstrate aberrant methylation of microRNA genes *miR-124*, *miR-203*, and *miR-375* in a series of 41 common hepatocellular carcinoma cases [20]. Our own systematic study of a range of liver tumors including fibrolamellar carcinoma identified several frequently hypermethylated microRNA genes (Albat and Lehmann, in preparation). In FLC, *hsa-mir-9-1* is hypermethylated in 3/15 cases (20%), in HCC without cirrhosis in 6/10 cases (60%). *Hsa-mir-9-2* is hypermethylated in 6/15 cases (40%) in FLC and in 6/10 cases (60%) in HCC without cirrhosis. For both microRNA genes there are no significant differences in hypermethylation between FLC and HCC without cirrhosis (Fisher's exact test, two-sided, p = 0.087 and 0.43, respectively) and the hypermethylation in both entities is quite frequent (20 to 60%).

### Absence of *APC*, *CDH1*, and *GSTπ1* gene hypermethylation in FLC

All specimens from patients with FLC (tumor and adjacent normal tissue) showed only little or no methylation in the *APC* and the *CDH1* promoter not qualifying for hypermethylation status according to the definitions outlined above.

On the contrary, 3 samples showed a clear reduction of the methylation levels of the *APC* gene in FLC compared to adjacent non-neoplastic tissue from the same patient.

The *GSTπ1* gene showed variable methylation levels in the FLC specimens (15–50%) but to a very similar extent in nearly all samples of surrounding normal liver tissue (10–55%). Therefore, following the stringent threshold defined above, no FLC sample qualifies as “hypermethylated”. Applying the cases-specific definition of hypermethylation (“50% more than in the paired normal tissue”, see above), 4/17 (23.5%) qualify as “hypermethylated” in the *GSTπ1* gene.

Since the *APC* gene and the *GSTπ1* gene were found to be hypermethylated in 60% (6/10 cases each) of common HCC differences between FLC and HCC groups were significantly different (p = 0.0009 for both genes, Fisher's exact test, two-sided).

### Global methylation level in FLC

Global loss of methylation is well described for many solid tumors and also for hepatocellular carcinoma [21]. Therefore, the methylation level of *LINE-1* sequences, highly repetitive DNA elements scattered throughout the human genome, was assessed using quantitative pyrosequencing. The methylation level of these elements correlates very well with the global methylation level of the genome under study and therefore can serve as surrogate marker for the overall methylation level [12]. In comparison to adjacent non-neoplastic liver tissue, no significant demethylation of *LINE-1* sequences was found in FLCs, indicating the absence of widespread hypomethylation in this liver tumor (**Figure 3**). In contrast, in common HCC from non-cirrhotic liver samples a significant decrease of *LINE-1* methylation in comparison to adjacent non-neoplastic liver tissue was identified (p<0.0001, **Figure 3**). The *LINE-1* methylation level in the non-neoplastic liver tissue from both patient groups (FLC and HCC) did not show any differences (81.6+/−7% versus 79.1+/−7%, p = 0.26, M-Whitney-U, two-tailed).

### Cluster analysis of methylation data

**Figure 4:**
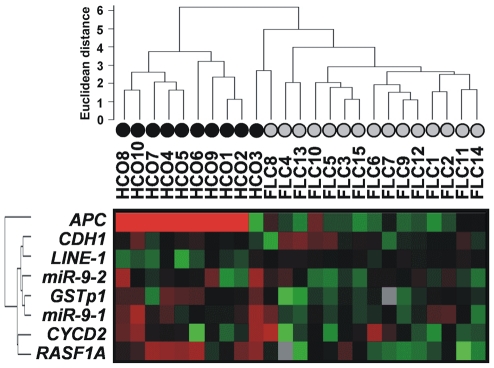
Cluster analysis of methylation data for FLC and common HCC without cirrhosis. The quantitative methylation data were log-transformed and translated into a color code.

Unsupervised clustering of all quantitative log-transformed methylation data revealed a very good separation of the FLC samples from the HCC samples (**Figure 4**). Within these two patient groups the separation between tumor and adjacent non-neoplastic tissue is less pronounced (see **Figure S2**). The clustering shown in **Figure 4** underlines the distinct methylation profile of FLC in comparison to common HCC arising in non-cirrhotic liver already apparent from **Figure 2**.

## Discussion

This is the first study that uses high-resolution quantitative methodology for comparison of gene-specific and global DNA methylation patterns of a large series of FLCs (n = 15) with those in common HCC arising in non-cirrhotic liver. Interesting differences between these two entities were revealed.

FLCs frequently harbor gene-specific hypermethylation in the absence of global hypomethylation. For several loci the frequency of aberrant hypermethylation is indistinguishable from conventional hepatocellular carcinoma arising in the non-cirrhotic liver, whereas for the *APC* and the *CDH1* gene statistically significant differences in hypermethylation could be found.

These data demonstrate that global reduction in DNA methylation and gene-specific hypermethylation appear to represent independent events during tumor evolution of FLCs. These observations are in accordance with studies focusing on common hepatocellular carcinoma [22] and also other epithelial tumors (e.g. prostate carcinoma [23] or urothelial carcinoma [24]), which also show that global and gene-specific hypermethylation are independent events. The data also demonstrate that full-blown malignancy (i.e., carcinoma) can develop in the absence of global hypomethylation.

Our results are also in line with the data from Kim et al. [25], who showed that in common HCC global hypomethylation can take place independent from cirrhosis.

The less distinct separation of FLC from adjacent tissue and HCC from adjacent tissue, respectively, supports the concept of “field cancerization” [26], which involves epigenetic field defects also in the non-neoplastic tissue adjacent to full-blown malignancy.

One FLC case was HBV positive (no. 13) and one common HCC case was HCV positive (no. 10). But in both cases careful reanalysis of all methylation data did not reveal any peculiarity in comparison to the hepatitis-negative cases (see also **Figure S1**).

The results presented in this study are partly at variance with another study of DNA methylation in a series of 5 FLCs describing low levels of methylation in all FLCs without any difference in comparison to adjacent non-neoplastic liver tissues [27]. We found unequivocal and frequent hypermethylation of several loci (e.g., *hsa-mir-9-1, hsa-mir-9-2, cyclinD2, RASSF1A*) as well as total absence of hypermethylation of the *CDH1* (E-cadherin) gene. By contrast, the *CDH1* gene is reported by Vivekanandan and Torbenson to be methylated in 4/4 normal liver samples and 5/5 FLC and 4/3 FLC metastases. In part, these discrepancies can be explained by selection of genes. microRNA genes and the *APC* gene were not included by Vivekanandan and Torbenson. However, the most likely reason is difference in detection methodology: Vivekanandan and Torbenson used exclusively conventional qualitative methylation-specific PCR (MSP). From Figure 1 in their publication it can be deduced that they scored any sample showing a PCR product using M-primers as “methylated”, regardless of band intensity and ratio between M- and U-band.

This explains for example the occurrence of 100% methylation of the *CDH1* gene in normal liver samples and the inability to detect differences in methylation levels between tumor specimens and adjacent non-neoplastic liver tissue. Employing the very same MSP primers for the analysis of all FLC specimens a weak “M-band” of variable intensity relative to the corresponding “U-band” was observed for 7 tumor and 6 adjacent tissue samples (see Figure S3).

Several groups (including our own) have demonstrated the importance of employing quantitative methods in studying aberrations in DNA methylation [6], [28], [29]. A mere qualitative analysis is not able to identify highly significant differences between tumor and surrounding tissue as well as between different tumor types.

### Conclusions

The results presented demonstrate the presence of frequent aberrant DNA methylation in fibrolamellar carcinoma. However, these differences were only discernible employing quantitative methodology. In comparison to common HCC arising in non-cirrhotic liver clear differences exist, separating these morphologically and clinically distinct entities also on the epigenetic level. The aberrant hypermethylation in FLC specimens takes place in the absence of a global loss of methylation, indicating that these two epigenetic aberrations are well separated phenomena and that full-blown malignancy can develop in the absence of global hypomethylation.

## Materials and Methods

### Patient material

Cases of FLC (n = 15) and HCC of non-cirrhotic liver (n = 10) from the period from 1988 to 2007 were retrieved from the archive of the Institute of Pathology, Medizinische Hochschule Hannover, Germany and analyzed anonymously. The local Ethics committee (“Ethik-Kommission der Medizinischen Hochschule Hannover”, head: Prof. Dr. Tröger) exempted this study from review because all specimens under study were retrieved anonymously and retrospectively (left-over samples from diagnostic procedures) and waived the need for consent due to the fact the samples received were anonymous. Age, sex, and TNM stage of the tumors were extracted from the histological reports (**see** **Table 1**). Clinical follow up data were not available. Cases were independently reviewed by two diagnostic histopathologists (PF, FP). Areas of tumor and non-tumor tissue were marked and DNA was isolated from unstained serial sections using these marked slides as guidance for manual microdissection.

### DNA extraction and bisulfite treatment

Genomic DNA was isolated from formalin-fixed paraffin-embedded specimens using proteinase K-digest over night (50 mM Tris pH 8,1; 1 mM EDTA; 0,5% Tween 20; 10 µg/ml proteinase K), followed by exhaustive organic extraction and ethanol precipitation. Subsequently, DNA samples were treated with sodium bisulfite using the EZ DNA Methylation Kit™ (Zymo Research, HiSS Diagnostics, Freiburg, Germany) following the manufacturer's instructions and finally eluted in 40 µL elution buffer.

### Generation of the PCR-products for methylation analysis

PCR products were generated in a 25 µL reaction volume with 400 nmol/L of forward, 40 nmol/L reverse and 400 nmol/L universal biotinylated primers, 200 µmol/L of each dNTP, 1.5 mmol/L or 2.5 mmol/L MgCl_2_ (see **Table 2** for all primer sequences and reaction conditions), 1× Platinum-Taq reaction buffer and 1.25 units PlatinumTaq™ (Invitrogen, Karlsruhe, Germany). PCR conditions were 95°C for 5 minutes, followed by 45 cycles with denaturation at 95°C for 30 seconds, annealing at 55°C or 60°C (see Table 1) for 45 seconds, and elongation at 72°C for 30 seconds finished with 1 cycle final elongation at 72°C for 5 minutes. The reverse primer is tagged by a sequence recognized by the universal primer. Therefore, a single (expansive) biotinylated primer can be used for all different gene-specific assays [30].

**Table 2:** Primer sequences.

Gene	Forward primer	MgCl_2_ [mM]	T_Ann_ [°C]	Size (bp)
***mir-9-1***	f: GGG AAA TGG GGTATT AGA AAT TTTr: [GGG ACA CCG CTG ATC GTT TA] CAA CAA CAA AAA CCT CAA ACA CPyro: TTT TTG GGT TTG GAT	1,5	60	140
***mir-9-2***	f: GGA AGA GAT GTT GAT TGA GAA AAr: [GGG ACA CCG CTG ATC GTT TA] TAA TCA ACC AAC TAC CCC ACPyro: GGG ATT GTT GTA ATG TTG	1,5	60	114
***APC***	f: GGA GAG AGA AGT AGT TGT GTA ATT Tr: [GGG ACA CCG CTG ATC GTT TA]A CTA CAC CAA TAC AAC CAC ATA TCPyro: TTA GGG TGT TTT TTA TTT T	2,5	55	123
***CDH1***	f: AGA TTT TAG TAA TTT TAG GTT AGA GGr: [GGG ACA CCG CTG ATC GTT TA]C TAA TTA ACT AAA AAT TCA CCT ACCPyro a: ATT TTA GGT TAG AGG GTT ATPyro b: TTT GGG GAG GGG TT	1,5	55	134
***Cyclin-D2***	f: GTA TTT TTT GTA AAG ATA GTT TTG ATTr: [GGG ACA CCG CTG ATC GTT TA] CCA AAC TTT CTC CCT AAA AACPyro: ATA GTT TTG ATT TAA GGA TG	1,5	55	117
***ESR1***	f: GGY GAG GTG TAT TTG GAT AGT AGr: [GGG ACA CCG CTG ATC GTT TA]C TAT TAA ATA AAA AAA AAC CCC CPyro: GTA TTT GGA TAG TAG TAA GTT	2,5	55	208
***GSTπ1***	f: GGG GAG GGA TTA TTT TTA TAA Gr: [GGG ACA CCG CTG ATC GTT TA]A ATT AAC CCC ATA CTA AAA ACT CTPyro: GGA TTA TTT TTA TAA GGT	2,5	55	173
***LINE-1***	n/a (Qiagen)	1,5	50	n/a
***MINT31***	f: GTT TAG GGG TGA TGG TTT TAGr: [GGG ACA CCG CTG ATC GTT TA]A AAC ACT TCC CCA ACA TC TACPyro: GTG GTG ATG GAG GTT AT	1,5	55	188
***RASSF1A***	f: AGT TTG GAT TTT GGG GGA GGr: 5′-Biotin-CAA CTC AAT AAA CTC AAA CTC CCCPyro: GGG TTY GTT TTG TGG TTT	1,5	60	136
***SFRP1***	f: TTG GGG ATT G**YG** TTT TTT GTTr: [GGG ACA CCG CTG ATC GTT TA] ACT CTA **CR**C CCT ATT CTC CPyro: GAG GTT TTT GGA AGT TTG	1,5	55	108
***SOCS1-a***	f: GTG AAG ATG GTT T**Y**G GGA TTTr: [GGG ACA CCG CTG ATC GTT TA]C AAC **R**AA ACC CCC AAC ATA CPyro: TT**Y** GAG TTG TTG GAG TAT TA	1,5	55	150
***SOCS1-b***	f: GTT TTT AG**Y** GTG AAG ATG GTT Tr: [GGG ACA CCG CTG ATC GTT TA] CTA AC**R** AAA CAA CTC CTA CAA CPyro: GTT TTT ATT TGG ATG GTA G	1,5	55	221
**univ-bio**	5′-Biotin-[GGG ACA CCG CTG ATC GTT TA]			

Y = Pyrimidine (C/T).

R = Purine (A/G).

f = forward primer.

r = reverse primer.

Pyro = pyrosequencing primer.

Sequences in square brackets resemble universal tag for biotinylated primer.

### Methylation analysis using Pyrosequencing

PCR products (5–20 µL) were added to a mix consisting of 3 µL Streptavidin Sepharose HP™ (Amersham Biosciences, Freiburg, Germany) and 37 µL binding buffer (Qiagen, Hilden, Germany) and mixed at 1200 rpm for 5 minutes at room temperature.

Using the Vacuum Prep Tool™ (Qiagen, Hilden, Germany), single-stranded PCR products were prepared following the manufacturer's instructions. The sepharose beads with the single stranded templates attached were released into a PSQ 96 Plate Low™ (Qiagen, Hilden, Germany) containing a mix of 12 µL annealing buffer (Qiagen, Hilden, Germany) and 500 nmmol/L of the corresponding sequencing primer (**see** **Table 2**). Pyrosequencing™ reactions were performed in a PyroMark MD System (Qiagen, Hilden, Germany) according to the manufacturer's instructions using the PyroGold SQA™ Reagent Kit (Qiagen, Hilden, Germany). CpG site quantification was performed using the new methylation Software Pyro Q-CpG™.

Criteria for Pyrogram™ selection were as follows: sufficient peak height of >25 units for a single nucleotide (arbitrary units for light emission calculated by the software), sharp symmetric peaks without any irregularities or side-peaks, and a wide reading length with a high reliability until the end of the sequence. Furthermore, the absence of any significant signals at the positions where a bisulfite treatment control was included or where control nucleotides were dispensed to check for unspecific background signals.

### Methylation analysis using conventional Methylation-specific PCR

For conventional qualitative methylation specific PCR the primer pairs described by Vivekanandan and Torbenson were used [27]. 20–50 ng bisulfite treated DNA were amplified using 0.5 units of Taq polymerase (Platinium Tag, Invitrogen, Karlsruhe Germany) in the presence of 200 µM dNTPs, 1.5 mM MgCl_2_ and 10 pmol of each primer in the reaction buffer provided by the manufacturer in a final volume of 25 µl. After an initial denaturation of 2 min at 95°C, 40 cycles consisting of 30 sec at 95°C, 30 sec at 65°C and 40 sec at 72°C followed.

The PCR products were resolved on a 6% PAA gel and visualized employing ethodium bromide staining.

### Statistical analysis

Statistical differences were calculated using the Mann-Whitney-U test. All calculations were performed using the software package GraphPad Prism (version 5.01 for Windows, La Jolla, CA, USA). p<0.05 were considered statistically significant.

For cluster analyses the quantitative methylation data from all samples were log-transformed and uploaded into the statistical package BRB array tool (version 3.5.0-Patch_2) [31]. Hierarchical clustering was then performed applying Euclidean distance as a dissimilarity metric and the complete linkage clustering method.

## Supporting Information

Figure S1 Comparison of the two different definitions of “hypermethylated” (see “Results”) if applied to all methylation measurements performed in this study.
(0.26 MB TIF)

Figure S2 Separate clustering of methylation data for FLC and adjacent non-neoplastic tissue (A) and common HCC from non-cirrhotic liver and adjacent non-neoplastic tissue (B).
(1.37 MB TIF)

Figure S3 MSP results for three tumor/adjacent tissue sample pairs using the primers described by Vivekanandan and Torbenson. FLC7 shows a weak “M-band” in the adjacent tissue, FLC8 in the tumor specimen and FLC4 in both fractions. Altogether 7 tumor and 6 adjacent tissue specimens displayed an “M-band” of variable intensity relative to the corresponding “U-band”.
(0.07 MB TIF)
