# Supplementary figures and images for: Frequent and Distinct Aberrations of DNA Methylation Patterns in Fibrolamellar Carcinoma of the Liver
Source: PLoS One. 2010 Oct 29;5(10):e13688. doi: 10.1371/journal.pone.0013688 (PMC2966398; doi:10.1371/journal.pone.0013688)

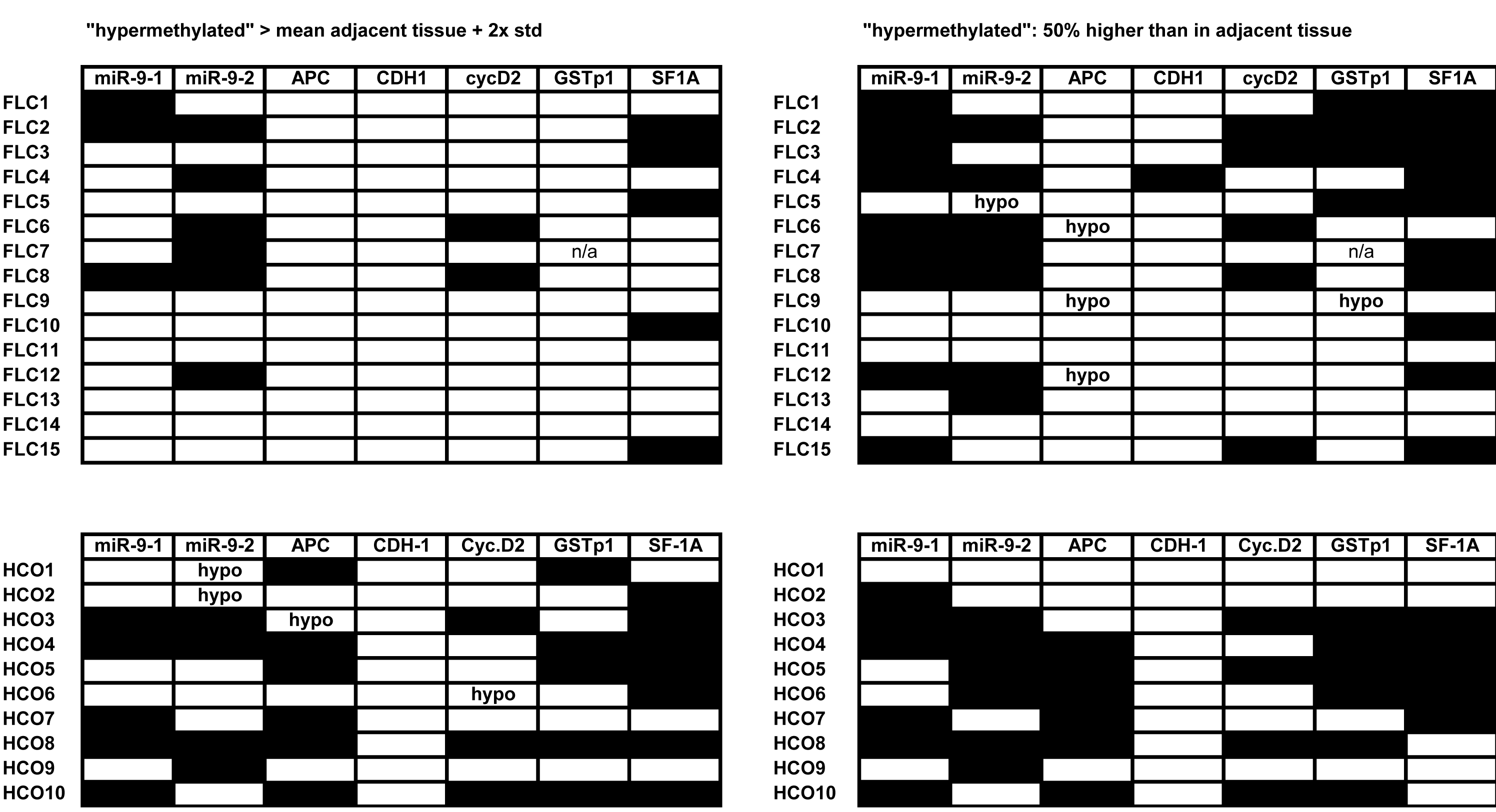

Supplement: Figure S1 — Comparison of the two different definitions of “hypermethylated” (see “Results”) if applied to all methylation measurements performed in this study. (0.26 MB TIF) [file pone.0013688.s001.tif]

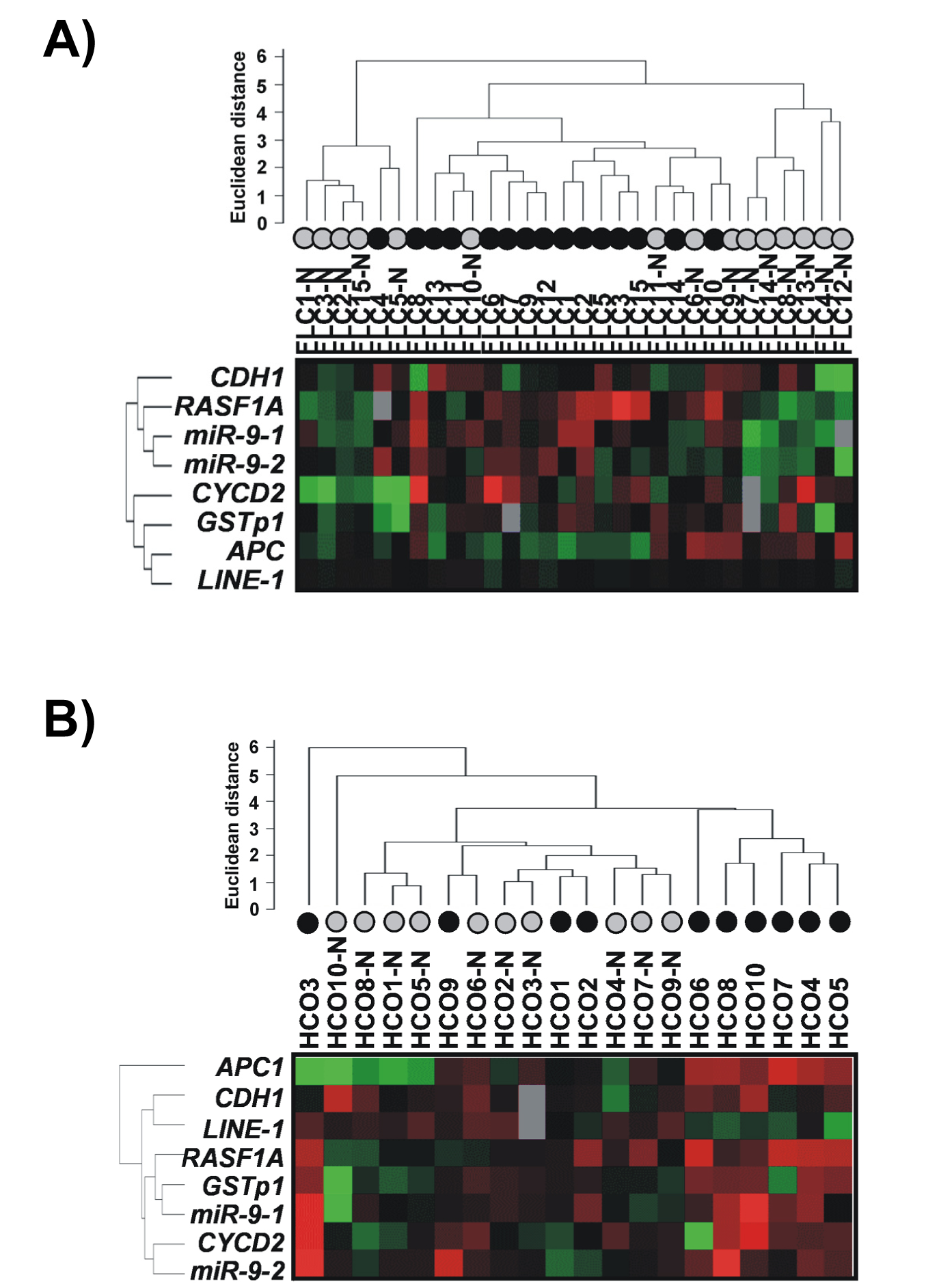

Supplement: Figure S2 — Separate clustering of methylation data for FLC and adjacent non-neoplastic tissue (A) and common HCC from non-cirrhotic liver and adjacent non-neoplastic tissue (B). (1.37 MB TIF) [file pone.0013688.s002.tif]

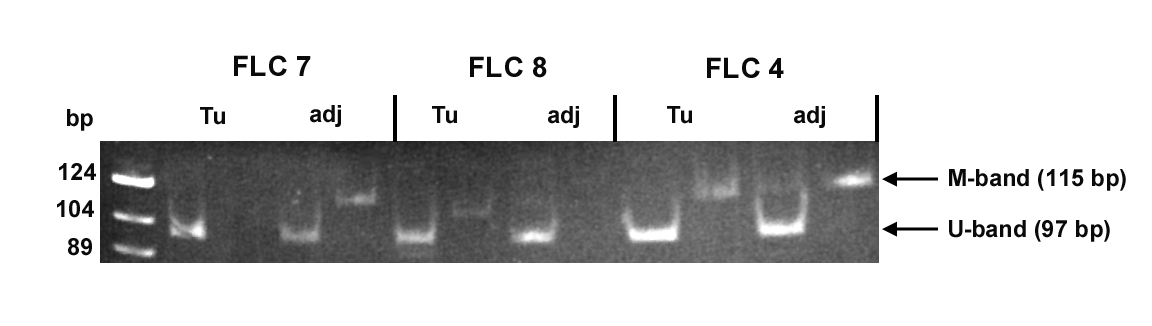

Supplement: Figure S3 — MSP results for three tumor/adjacent tissue sample pairs using the primers described by Vivekanandan and Torbenson. FLC7 shows a weak “M-band” in the adjacent tissue, FLC8 in the tumor specimen and FLC4 in both fractions. Altogether 7 tumor and 6 adjacent tissue specimens displayed an “M-band” of variable intensity relative to the corresponding “U-band”. (0.07 MB TIF) [file pone.0013688.s003.tif]
